# Little if any role of male gonadal androgens in ontogeny of sexual dimorphism in body size and cranial casque in chameleons

**DOI:** 10.1038/s41598-020-59501-6

**Published:** 2020-02-14

**Authors:** Anna Bauerová, Lukáš Kratochvíl, Lukáš Kubička

**Affiliations:** Faculty of Science, Charles University, Department of Ecology, Viničná 7, 128 43, Prague 2, Czech Republic

**Keywords:** Animal physiology, Herpetology, Bone

## Abstract

Proximate control of the development of sexual dimorphism is still hotly debated in reptiles. In some squamates, many male-typical exaggerated traits including body size were assumed to be controlled by masculinization by male gonadal androgens. We performed a manipulative experiment to test the importance of this mechanism in the development of pronounced sexual differences in body size and size of head casque in the chameleon *Chamaeleo calyptratus*. Castrated males attained male-typical body size highly deviating from the body size of control females. Ontogenetic allometries of casque size on head length revealed that sexes depart considerably in casque growth later in the ontogeny; however, castrated males still follow male-typical casque growth. Paradoxically, exogenous testosterone led in females to slight increase of casque size, which might reflect interference with the feminizing effects of female gonadal hormones. The results in males strongly suggest that masculinization by male gonadal androgens during growth is not required for the development of sexual dimorphism in body size and casque size in the chameleon. The ontogeny of sexually dimorphic body size and exaggerated traits in at least some squamates is likely controlled by other proximate mechanism, possibly by feminization by ovarian hormones.

## Introduction

Body size is one of the most prominent sexually dimorphic traits in animals. Squamate reptiles, an important group of vertebrates with a huge variability in body size, represent no exception. In many lineages of squamates, even closely related species may differ in the magnitude and also in the direction of sexual dimorphism in body size^1–4^. Despite several studies, the proximate mechanism allowing this notable evolutionary plasticity in sexual dimorphism in body size is still poorly understood and several hypotheses on the proximate mechanisms responsible for the ontogeny of sexual dimorphism in body size have been suggested for squamates^2–7^.

The most hotly debated is the male androgen hypothesis suggesting that male growth is masculinized by gonadal androgens. Sex-specific levels of gonadal steroids are generally important for the control of sexually dimorphic traits in vertebrates^8^ and the expression of many male-typical morphological, physiological and behavioural traits has been linked to the levels of male gonadal androgens present in squamates (e.g., size of copulatory organs^9^, activity of scent glands^10^, colouration^11^, odour and other sex recognition cues^12^, social behaviour^13,14^). Nevertheless, the evidence that the growth of rigid, skeletal structures, for example total structural body size, is solely under the control of male gonadal androgens, is equivocal. Cox *et al*.^3^ presented broader evidence for the bipotential effect of gonadal androgens which assumes that male gonadal androgens *masculinize* growth with a growth-stimulating effect in male-larger species and a growth-suppressing effect in female-larger species^2,3^. This hypothesis was supported mainly by experiments on members of the lizard group Iguania. However, the results in gekkotan lizards question the generality of this hypothesis: castration had no significant effect on male final body size and growth trajectory in male-larger^7,15,16^ as well as in female-larger geckos^17^. The discrepancies in the support for the male androgen hypothesis between geckos and other squamates reported by Cox *et al*.^3^ could be explained by lineage-specific differences in the regulatory function of male androgens between iguanian and gekkotan lizards, as suggested by Pollock *et al*.^18^. Alternatively, they can reflect differences in experimental procedures. In Iguania the support for the male androgen hypothesis largely came from short-term growth experiments without a control of the social environment. Also, the comparison of growth between control males and males treated with exogenous testosterone (T) was often taken as a support for the hypothesis. The results of the comparison of control males and castrated males, the stronger test of the effects of male gonadal androgens not supporting the hypothesis, were often not taken into account (reviewed in Starostová *et al*.^15^). The male androgen hypothesis received support from the results of the manipulative experiment in squamate females treated with exogenous androgens^15,17,19^. However, exogenous androgens in females can lead to male-typical growth via the interference with normal hormonal secretion of ovaries and hence causing *defeminisation*, i.e. the suppression of the development of female-typical morphology, rather than masculinization presuming the direct effect of androgens on growth^7,15^. Supporting evidence is found in geckos, where females treated with exogenous testosterone possessed underdeveloped ovaries^15^ and ovariectomized females follow male-typical growth trajectories even without application of exogenous androgens^7^. To test whether two major lizard lineages (Gekkota and Iguania) differ in the key growth regulator responsible for the ontogeny of sexual dimorphism in body size, we performed a long-term manipulative growth experiment using the veiled chameleon (*Chamaeleo calyptratus*), the highly sexually dimorphic member of the lineage Iguania, under controlled conditions. Alongside the hormonal control of sexual dimorphism in structural body size, we also investigated the ontogeny and hormonal control of sexual dimorphism in casque size. Some lineages of chameleons are known for notable sexual dimorphism in the presence and size of head structures such as horns, gular appendages, occipital lobes and casques. These structures have attracted the attention of evolutionary biologists ever since Darwin, who (misled by incorrect information he received from Dr. Günther that chameleons are “peaceable creatures” and it is thus hardly believed that they “would ever become pugnacious”) interpreted “almost monstrous deviations of structure” on chameleon heads as masculine ornaments^20^. Now, we are aware that male-male combat is very frequent in chameleons and recent evidence documents that bite force is highly related to head size in chameleons^21^. A rugose cranial casque supported by bony protrusions is highly sexually dimorphic in many chameleons including *C. calyptratus*. High casques provide an expanded area for muscle attachments^22^, which can increase bite force. Casque size thus might be an honest signal of fighting ability^21^. We followed the ontogeny and hormonal control of casque size in the veiled chameleon to test whether this primarily bony structure shares proximate control with other bones or whether this putative honest intrasexual signal of fighting ability has different hormonal control and is tightly linked to male androgen levels as other such traits (although not so rigid) in other vertebrates (e.g., birds^23^, mammals^24^).

In our set up, we tested the effect of castration with and without T replacement via application of exogenous T in males and the effect of the application of exogenous T in females on growth curves, final body size and casque size. This design allows us to test the contrasting hypotheses on the control of the growth of skeletal structures. The male androgen hypothesis predicts that the removal of gonads in males will demasculinize their growth and that the effect will be reversed by the application of exogenous T in male castrates. In females, this hypothesis predicts masculinization of growth after treatment by exogenous T. On the other hand, the ovarian control hypothesis predicts that gonadal removal and castration followed by the application of exogenous T will have little effect on structural growth in males, while exogenous T will defeminize growth in females.

## Materials and Methods

The veiled chameleon, *Chamaelo calyptratus*, is an arboreal lizard native to southern Arabia^25^. It is a popular pet and has also become a well-studied laboratory reptile for physiology, developmental biology and behavioural ecology^26–31^.

The experimental animals were obtained from a private breeder at the age of two days. They were the progeny of two related females. We housed them individually in standardized plastic boxes containing tree branches for climbing. The ambient temperature ranged between 23 to 26 °C with a basking spot of around 34 °C produced by UVB bulbs (Exo Terra Reptile UVB100) during the light phase of the day (12 hours). The chameleons were fed to satiety every day with crickets (*Gryllus assimilis*) dusted with vitamins (Roboran, Univit, Olomouc, Czech Republic) and calcium (Vitacalcin, Zentiva, Prague, Czech Republic).

We determined the sex of individuals according to tarsal spur, a male-typical sexually dimorphic trait which is visible in males already at the time of hatching although this species generally reach sexual maturity not sooner than at sixth month of age^30^. At the age of one month we established three groups of males and two groups of females. Each group was balanced with respect to body mass and head length. Before any surgery or experimental manipulation, we assigned each group randomly to three treatments in males and two in females: Control males (sham-operated), Testosterone males (castrated males treated with T), Castrated males, Control females (sham-operated virgin females) and Testosterone females (sham-operated virgin females treated with T).

According to experimental design, the following surgery was performed in all (40) experimental animals, i.e., 8 individuals per treatment group, at the very young age of 40 to 43 days, which was long before reaching final body size or sexual maturity (see e.g. the arrow pointing to the time of surgery in the Supplementary Fig. 1, where the whole growth trajectory of all experimental animal is depicted). Prior to surgery, animals were anaesthetized by intramuscular injection of ketamine (Narkamon 5%, Spofa a.s., Prague, Czech Republic; applied twice in 15 min. intervals, together 300 μg/g of body mass) combined with hypothermia. The gonads were exposed via a lateral incision. Bilateral orchiectomy was performed on Castrated males and Testosterone males by ligating each testis with surgical silk, then ablating and removing it. For the remaining groups, “sham” surgeries were performed, where the gonads were exposed via incision but remain intact. The incision was closed using Prolene surgical suture (Ethicon INC, Somervile NJ, USA) and covered with Glubran 2 surgical glue (GEM S.r.l., Viareggio, Italy). The stitches were removed within two weeks after the wound had healed sufficiently and at this time the cutaneous application of oil-diluted T commenced in groups with T treatment^7,12–14^. Briefly, 0.25 μg of crystalline T (Sigma Aldrich) per gram of body mass dissolved in pharmaceutical quality sunflower oil was applied to the skin on the back of the casque of each experimental individual twice a week at regular intervals (every 3 to 4 days). The mixture was absorbed into the skin within several hours. At the same time, pure sunflower oil was applied in the same way to Control males, Control females and Castrated males. Experimental animals were weighed twice a week for the mass-specific estimation of hormonal or placebo dosage. In total, hormonal manipulation or placebo treatment proceeded 82 following weeks.

Head measurements i.e., head length (HL, measured from the tip of the snout to the mandibular joint), head-casque height (HCH, measured from the lowest part of the mandibula to the top of the casque) and head height (HH, measured from the lowest part of the mandibula to the superciliary arc) were taken using callipers every two weeks during the period of the fast growth (first 26 weeks), then every four weeks. Not to stress the animals with excessive manipulations too often, the leg length (LL, length of hind tibia and tarsus) was measured every second week during the period of fast growth (first 26 weeks) and later again from the 52^nd^ week of age in four-week intervals. Snout-vent length is often used as a measure of structural body size in squamates; however, it was not included here due to being extremely difficult to achieve in chameleons, especially in larger individuals. All Control females and Testosterone females laid clutches of unfertilized eggs during the experiment. Chameleons have large clutch sizes^32^ causing dramatic fluctuations in body mass according to the reproductive stage in experimental females and so we did not include growth in body mass in our analyses. The growth experiment was terminated within the 89^th^ week of age, long after the growth of all experimental animals had slowed considerably, which was observed *c*. 50 weeks after surgery (e.g., Supplementary Fig. 1). Prior to the last measurement, the social behaviour of the animals was recorded for another project (up to eight 20 minute interactions per individual).

Originally, each treatment group consisted of 8 individuals. Unfortunately, some animals died before the end of the growth experiment. Seven of these (i.e., one Control male, two Testosterone males, one Castrated male, one Control female and two Testosterone females) died suddenly from unknown reason within the age of 18 to 48 weeks. We found no differences in growth rate when compared to the living members of given experimental group and we observed no indication of reduced viability before they deceased. However, these seven prematurely deceased animals were excluded from all statistical analyses. After their exclusion, experimental groups did not differ in body mass (mean of 4.61 grams) and LL (mean of 10.81 mm), taken at the time of surgery (ANOVA: F_4,28_ < 0.71, p > 0.59 for both cases) but were significantly different in HL (ANOVA: F_4,28_ = 2.97, p = 0.036) which was slightly larger in Control males (mean of 12.96 mm) than in Testosterone males (mean of 12.05 mm) and Testosterone females (mean of 11.63 mm) (Post-hoc Fisher tests: p < 0.029 for comparisons between these groups). In all other groups, HL was comparable at the time of surgery (Post-hoc Fisher tests: p > 0.07), as the mean HL for Control females was 12.26 mm and for Castrated males 12.31 mm. The relative head shape represented either by HCH or HH with HL as a covariate, was comparable among all treatment groups at the time of surgery (full-factorial ANCOVA: differences neither in interaction, nor in factor group, F_4,23_ < 1.12, p > 0.37 in all cases).

Another five animals (two Control males, one Testosterone male and two Control females) died relatively close to the final measurement (at the age of 63–72 weeks). Veiled chameleons are relatively short-living reptiles with average life span of two years^30^. All these five animals died long after they exceed the first year of life. Their growth also did not depart from the common trend for given experimental groups (Supplementary Fig. 1). Therefore, due to all circumstances, their death can be tentatively attributed to the natural senescence. Moreover, as the original growth data were already sufficient for the analyses we were able to estimate asymptotic HL and LL and, with one exception (one Testosterone male), we were also able to collect their blood plasma for subsequent treatment validations. The experimental treatment of this Testosterone male was verified by behavioural assay with female stimulus, where he followed the behaviour of other males with high Testosterone level. Namely, this male performed courtship behaviour towards stimulus female while none of castrated males did (own unpublished data). The final number of experimental animals in each experimental group was: seven Control males, six Testosterone males, seven Castrated males, seven Control females and six Testosterone females.

Prior the end of the experiment, we took blood plasma from the tail blood vessel in each of the experimental individuals for hormonal treatment verification. Circulating plasma levels of estradiol (E_2_), androstenedione (AD), dihydrotestosterone (DHT) and T were measured at the Institute of Endocrinology (Prague, Czech Republic). Liquid chromatography-tandem mass spectrometry (LC-MS/MS) after Sosvorova *et al*.^33^ was used for the detection of AD and T. Therefore, the results should not be biased by possible cross-reaction with other androgens^34^. Briefly, the method consists of plasma extraction with diethyl ether followed by the appropriate derivatization step (to enhance detection responses of steroids in the MS) and separation using the ultra-high liquid chromatography Eksigent ultraLC 110 system (Redwood City, CA, USA). Detection of analytes was performed on an API 3200 mass spectrometer (AB Sciex, Concord, Canada) with the electrospray ionization probe operating in a positive mode. Their quantification was determined using calibration curves based on known analyte concentrations. The limits of detection were 0.01 ng per ml. For DHT, the standard radioimmunoassay (RIA) protocol after Hampl *et al*.^35^ was used. The method consists of extracting plasma with diethyl-ether followed by a radioimmunoassay using rabbit polyclonal antiserum to dihydrotestosterone-7-(carboxymethyloxime) bovine serum albumin conjugate, and [^3^H]DHT. Selective oxidation with potassium permanganate was applied to the sample to eliminate T to prevent cross-reaction with the antiserum. Intra-assay and inter-assay coefficients of variation for the analyses are typically 17.1% and 17.7%, respectively. The limit of detection of the assay was 0.001 ng per ml. E_2_ was assayed using a commercial estradiol RIA kit A21854 (LOT No. 180607) from Beckman Coulter, Brea, California, USA, with a declared detection limit of 0.006 ng per ml. As the levels of hormones were measured in three different ways (one LC-MS/MS, two RIAs), it required a relatively high volume of blood plasma. Therefore, in several cases, the whole plasma volume obtained from a single individual was not sufficient to perform E_2_ measurements.

Because lizards are characterized by indeterminate growth (i.e., growth does not stop after sexual maturation), the reliable estimation of sexual dimorphism in body size independent from the effects of age requires knowledge of growth curves in both sexes^1^. For comparison of the final size among groups, we used asymptotic size in HL and LL estimated from the von Bertalanffy asymptotic growth model:

$$L=a(1-{e}^{-k(t-{t}_{0})})$$

where *a* is the asymptotic length L, *e* is the base of the natural logarithm, *k* is the rate of approach to asymptotic length, *t* is age (in days), and *t*_0_ is the hypothetical time at length zero. This model has previously been used to successfully describe the growth pattern in many lizards^1,7,16,17,36,37^. As asymptotic size is an estimation based on fitting the growth curve to multiple measurements, it is much less sensitive to errors of a single measurement. Moreover, using asymptotic size allows the comparison of animals of different ages, which in our case permitted the inclusion of five individuals which died shortly before the end of the experiment. The Shapiro-Wilk test was applied to detect any departure from normal distribution. When the null hypothesis of normal data distribution was rejected at α = 0.05, non-parametric tests were used. We used one-way ANOVA for comparison of values of asymptotic HL among treatment groups and successive Post-hoc Fischer LSD tests to reveal differences between treatment groups. Values of asymptotic LL and hormone levels departed from normality, therefore, Kruskal-Wallis ANOVA with Post-hoc Conover pairwise multiple comparison test were used for the comparison among groups. In addition, we compared static allometries in relative head shape among groups using full-factorial ANCOVA with either HCH or HH as the dependent variable, HL as the continuous predictor (covariate) and group identity as the categorical predictor. We also analysed differences in ontogenetic allometries in casque size (expressed by HCH) and HH on HL between groups by the linear mixed-effects model to account for repeated measurements of the same individual. The individual identity was built into the null model as the random effect to account for individual differences in intercepts of the ontogenetic allometries. We computed several models by adding to the null model treatment group as a fixed factor, HL only as a fixed continuous predictor, both group and HL as fixed predictors, and both group and HL and their interaction as fixed predictors. We compared the fit of these models using ANOVA and Akaike information criterion (AIC). When ΔAIC was <2, the models were considered equivalent and we selected the one with the less variables as the preferred model, while the more complex version was considered supported when ΔAIC > 2^38,39^.

Statistical analyses were conducted using Statistica version 10.0 (StatSoft, Tulsa, USA), lme4 modul^40^ implemented in R project^41^, and BrightStat.com (©Daniel Stricker and scians, GmbH Switzerland). Graphs were prepared using GraphPad Prism (version 6.07; GraphPad Software, San Diego, USA) and MS Excel (MS office 365+).

We followed the national guidelines for the care and use of animals. The experiment was conducted with the approval of the Ethical Committee of Charles University and the Central Commission for Animal Welfare and the environment of the Czech Republic (permit number 10803/2016-2).

## Results

The hormone assays validated treatment of all examined individuals (Table 1; Supplementary Fig. 2). In some cases, the hormonal levels were below the detection limit. In comparisons of hormone levels among treatment groups, we assigned these cases with the value of the limit of detection for a given hormone. The T and DHT plasma levels differed significantly among treatment groups (Kruskal-Wallis ANOVA: T: H_4,N=32_ = 27.90, p < 0.001; DHT: H_4,N=32_ = 26.63, p < 0.001; Table 1; Supplementary Fig. 2A,B). T and DHT levels of Testosterone males and Testosterone females were the highest and comparable between these two groups (Post-hoc Conover test: p > 0.05). All other groups possessed significantly different levels of T and DHT (Post-hoc Conover test: p < 0.05 in all cases) with Control females having the lowest T levels. There were also significant differences among treatment groups in AD (Kruskal-Wallis ANOVA: H_4,N=32_ = 25.92, p < 0.001; Table 1; Supplementary Fig. 2C). The highest and comparable AD levels were in Testosterone males and Testosterone females (Post-hoc Conover test: p > 0.05), the lowest and comparable levels were in Castrated males and Control females (Post-hoc Conover test: p > 0.05) and intermediate in Control males (Post-hoc Conover test: p < 0.001 in all cases). All remaining comparisons among groups significantly differed (Post-hoc Conover test: p < 0.001 in all cases). The plasma levels of E_2_ did not significantly differ among treatment groups (Kruskal-Wallis ANOVA: H_4,N=27_ = 6.15, p = 0.188; Table 1; Supplementary Fig. 2D). Here, however, the sample size was smaller, as there was not enough plasma to accurately measure this hormone in five animals (two Control males, one Castrated male, one Control female and one Testosterone female). Nevertheless, females possessed higher E_2_ levels than males regardless of experimental treatment when the animals were grouped according to sex (Mann-Whitney U test: U = 44.00, p = 0.032). Despite the hormonal treatment, Control and Testosterone females laid one to five clutches of unfertilized eggs. Both female treatment groups did not differ in mean number of clutches laid during the experiment (t-test: t = −0.61; p = 0.556).

**Table 1:** Summary of androstenedione, dihydrotestosterone, estradiol and testosterone plasma levels in experimental veiled chameleons (*Chamaeleo calyptratus*, Chamaeleonidae, Iguania).

	Androstenedione (ng ml^−1^)	Dihydrotestosterone (ng ml^−1^)	Estradiol (ng ml^−1^)	Testosterone (ng ml^−1^)
Treatment group	N, median, range	N, median, range	N, median, range	N, median, range
Control males	7, 0.635, 0.473–1.620	7, 1.098, 0.543–2.067	5, 0.255, 0.151–0.466	7, 43.70, 40.90–54.0
Testosterone males	5, 2.290, 2.010–5.450	5, 2.016, 1.135–3.936	5, 0.249, 0.083–0.353	5, 62.10, 56.70–72.60
Castrated males	7, 0.098, 0.010–0.675	7, 0.167, 0.053–0.327	6, 0.196, 0.072–0.277	7, 2.990, 1.260–20.90
Control females	7, 0.057, 0.010–0.198	7, 0.039, 0.012–0.067	5, 0.554, 0.042–1.002	7, 0.563, 0.010–2.280
Testosterone females	6, 2.035, 1.220–6.080	6, 2.073, 1.255–4.133	6, 0.471, 0.221–0.914	6, 58.850, 44.00–63.10

Range, minimal and maximal values of each hormone are presented. Original individual data and statistically homogenous groups are presented in the Supplementary Fig. 2.

The asymptotic von Bertalanffy model applied to the original data explained 94.4 to 99.2% of variability for HL and 95.2 to 99.9% for LL in each individual, demonstrating the applicability of this growth model to both body-size measurements. The asymptotic HL did not statistically differ from HL at the final measurements (t-test for dependent samples: t = 2.00; p = 0.054) and the same was true also for LL (Wilcoxon matched pairs test: Z = 0.49, p = 0.62) showing that the animals had already reached the size close to their asymptotic size.

**Figure 1:**
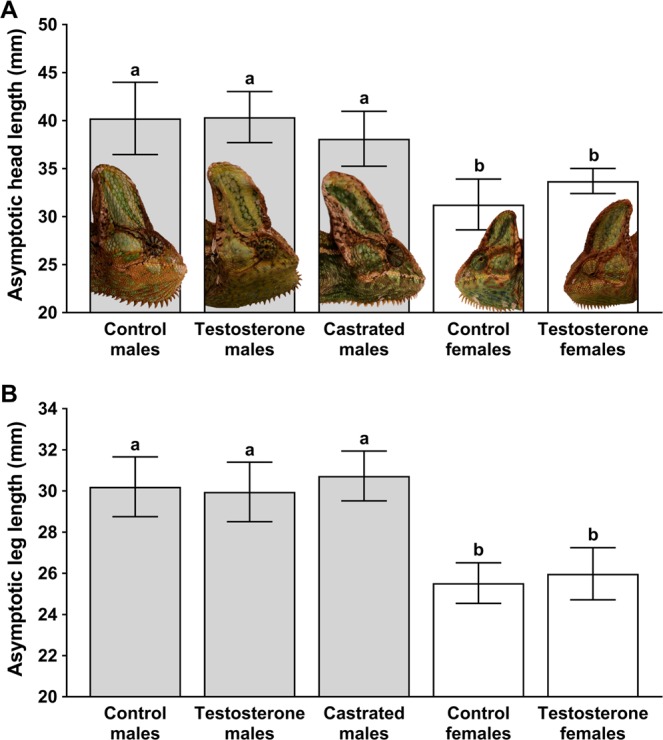
(**A**) Asymptotic head length and (**B**) asymptotic leg length in experimental animals of *Chamaeleo calyptratus*. Testosterone males denote testosterone-treated castrated males, Testosterone females denote testosterone-treated females. Means and 95% confidence intervals are given. Letters and column colour pattern denote statistically homogenous and non-homogenous groups. Head illustrations in the same scale in fully grown representatives from each experimental group are depicted.

The treatment groups differed significantly in the asymptotic HL (ANOVA: F_4, 28_ = 12.59, p < 0.001; Fig. 1A). The experimental males of each three groups reached comparable asymptotic HL (Post-hoc Fisher test: p > 0.18 for comparisons between these groups) and their heads were significantly longer than in both female groups (Post-hoc Fisher test: p < 0.013 in all comparisons). Control females and Testosterone females attained comparable asymptotic HL (Post-hoc Fisher test: p = 0.15). Similarly, asymptotic LL differed significantly among treatment groups (Kruskal-Wallis ANOVA: H_4,N=33_ = 22.60, p < 0.001; Fig. 1B). Asymptotic LL was comparable among the three male treatment groups (Post-hoc Conover test: p > 0.05 for all cases) and between female treatment groups (Post-hoc Conover test: p > 0.05). In intersexual comparison, all male treatment groups always possessed longer legs than both female treatment groups (Post-hoc Conover test: p < 0.001 in all intersexual comparisons; Fig. 1B). Although we are aware that our sample sizes are relatively small, we should keep in mind that we are testing large effects reflecting pronounced differences in size between sexes. On average, Control females were 30% and 18% smaller in asymptotic HL and LL, respectively, in comparison to Control males. Castrated males were comparable in size to Control males being on average 6% smaller in the asymptotic HL and 2% larger in the asymptotic LL (Fig. 1). These size differences between Castrated males and Control males could differ significantly when larger sample sizes are considered; however, for the test of the masculinization hypothesis, there is important that these male groups are comparable in size and both are much larger than Control females. Planning the experiment, we knew that there is a very large sexual size dimorphism in the studied species with males being around 1/3 larger in linear dimensions in comparison to females. Our question was whether the manipulation with androgens would change the final body size, most importantly whether the castration would shift males to female growth trajectory, or whether castrated males would keep typical male growth, i.e. we tested a rather large effect. We selected so highly dimorphic species of chameleons to robustly test this large effect using even small sample size. The *post hoc* analysis of the effect size confirmed that our sample size is adequate for testing sexual dimorphism and its ontogeny in final body size in this species. Cohen’s *d* (>2.5) computed from our data using means, standard deviations and sample size for Control males and Control females in asymptotic head length (our proxy of body size) points to a large effect indeed and the estimated minimum sample size for getting significant results for testing two-tailed *t*-test between these two groups is eight individuals (four in each group). Given the large differences between males and females, our sample size (*n* = 14, i.e. seven in each group) is adequate to reach statistically significant differences at the levels of α = 0.05 and β = 0.8. Similar differences were observed in leg length, our alternative measure of body size. Therefore, it is not surprising that the differences between Control males and Control females were significant in our ANOVA analyses as we expected during designing of our experiment. At that time, we also assumed based on the results of long-term growth experiments in other lizards^15–17^ that the effect of castration would be small. The major test of the male androgen hypothesis is the comparison between Control males and Castrated males, comparison among all other groups are not so important. There was a risk that the effect of castration would not be so small as we expected and that the results would be difficult to interpret given our small sample size. However, the results confirmed that the effect of castration is indeed very small. Based on the calculation of effect size (Cohen’s *d*) between Control males and Castrated males in asymptotic head length, one would need a minimum sample size of 94 individuals (47 in each group) at α = 0.05 and β = 0.8 to reach a significant result in two-tailed *t*-test between these two groups. Moreover, mean in the asymptotic leg length is higher in Castrated males than in Control males, which points that the differences in means are likely random and strongly supports the interpretation that the effect of castration on structural body size is negligible. Based on the *post hoc* statistical analysis of the effect size, one would have to repeat our experiment with at least 250 individuals, likely much more, to reach statistically significant differences between Control males and Castrated males in the asymptotic leg length. However, these differences would be still small and would change little in the interpretation that Control males and Castrated males grow to very similar size. Our interpretation that male gonadal androgens are not responsible for the large differences in body size between male and female chameleons are thus robust even with the small sample size. The robustness is not based on non-significant differences between Control males and Castrated males, but on the negligible effect of the castration on size.

**Figure 2:**
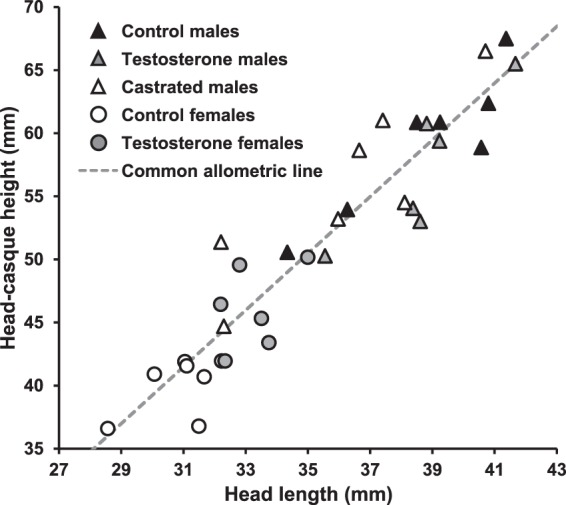
Static allometry in head-casque height relative to head length in fully grown individuals of *Chamaeleo calyptratus*. Solid triangles represent Control males, grey triangles Testosterone males (testosterone-treated castrated males), open triangles Castrated males, open circles Control females and grey circles Testosterone females (testosterone-treated females). Common allometric line is depicted.

**Figure 3:**
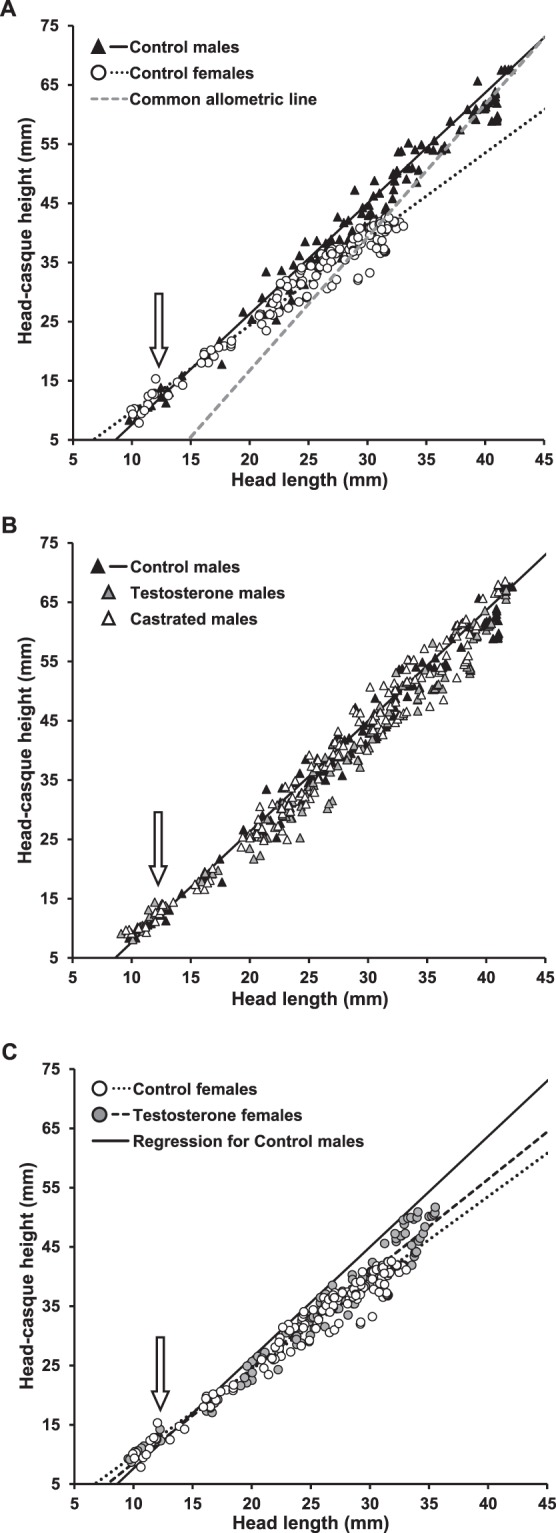
Ontogenetic allometry in head-casque height relative to head length in *Chamaeleo calyptratus*. Solid triangles represent Control males, grey triangles Testosterone males (testosterone-treated castrated males), open triangles Castrated males, open circles Control females and grey circles Testosterone females (testosterone-treated females). Arrows signify mean head length at surgery. (**A**) Comparison between Control males and Control females; line, solid for Control males and dotted for Control females, represents the regression with parameters from linear mixed-effects model accounting for repeated measurements of the same individual; grey dashed line represents the static allometry for all experimental animals for comparison. (**B**) Comparison between Control males (solid line, triangles), Testosterone males and Castrated males. (**C**) Comparison between Control males (solid line), Control females and Testosterone females; line, dotted for Control females and dashed for Testosterone females, represents the regression with parameters from linear mixed-effects model accounting for repeated measurements of the same individual.

At the time of the cessation of growth (56^th^ week after surgery, Supplementary Fig. 1), head shape, represented by HCH with HL as a covariate, was comparable among all treatment groups (full-factorial ANCOVA: neither interaction, nor group were significant, F_4,23_ < 0.51, p > 0.73 in both cases, Fig. 2).The similar head shape among groups was also found when HH (full-factorial ANCOVA: neither interaction, nor group were significant, F_4,23_ < 0.47, p > 0.76 in both cases) instead of HCH was used. However, ontogenetic allometries in HCH relatively to HL revealed a very different pattern. Control males and Control females differed greatly in the relationship between HL and HCH during ontogeny, with the males demonstrating much steeper ontogenetic allometry (mixed model ANOVA comparison of all models: differences in slope: p < 0.05, ΔAIC = 108.6; Fig. 3A). Castrated males and Testosterone males were similar in their ontogenetic allometries of casque size to Control males (mixed model ANOVA: differences in slope and intercepts between groups: p > 0.10, ΔAIC < 2.0; Fig. 3B), while Testosterone females possessed significantly steeper ontogenetic allometry in HCH than control females (mixed model ANOVA: differences in slope: p < 0.05; ΔAIC = 18.3), albeit not as steep as Control males (mixed model ANOVA: differences in slope: p < 0.05; ΔAIC = 48.7; Fig. 3C). There were no significant differences among treatment groups in the ontogenetic allometry of HH on HL (both group and HL-group interaction/ both group and HL, and group and HL and their interaction were not significant in mixed model ANOVA; ΔAIC < 2) supporting that the above differences in HCH reflect casque size.

## Discussion

Our manipulative growth experiment carried out with the veiled chameleon, *Chamaeleo calyptratus*, does not support the male androgen hypothesis for both final body size and relative casque size. Castrated males and Control males (as well as Testosterone males, but this group is much less relevant for the test) reached male-typical final body size in both proxies of structural body size (asymptotic length of head and hind limb, Fig. 1) and follow the same static (Fig. 2) and especially ontogenetic allometries in casque size relative to head length (Fig. 3B). These results are congruent with previous outcomes of structural body size in similar experiments carried out on geckos^15–17^ suggesting that ontogeny of sexual dimorphism in body size in at least some members of Gekkota and Iguania, two major clades of squamate reptiles, is not controlled by male gonadal androgens. As T is a precursor of both DHT and estrogens, T could affect phenotypic traits either directly via androgen receptor binding of T (and more efficiently DHT), or indirectly via metabolization of T into estrogens^42^. We observed elevated DHT levels in animals which also possessed elevated T levels (Table 1, Supplementary Fig. 2B). These differences still had no effect on the asymptotic size of the animal (Fig. 1), the results thus also ruling out a direct effect of male gonadal androgens on development of sexual dimorphism in body size, although it was recently suggested in a female-larger member of the clade Iguania^18^. It is quite surprising that experimentally increased T levels in both males and females accompanied the elevation of AD levels (Table 1, Supplementary Fig. 2C). AD, commonly produced in adrenals, is largely considered as a precursor of either testosterone or estrone^41^. Higher levels of AD in T-treated chameleons can reflect positive feedback between gonadal and adrenal androgens. Adrenal hyperandrogenism caused by androgen treatment has also been observed in mammals^43^. However, adrenal hyperandrogenism in chameleons did not affect their growth as well.

Previous studies on squamates documented that exogenous T leads to male-typical growth in females in both male- and female-larger species^15,17,19^. The male-typical growth of T-treated females was interpreted as masculinized by exogenous T under the male androgen hypothesis, but as defeminized under the ovarian control hypothesis^7,15^. It was shown that T-treatment in gecko females leads to ovarian shrinkage which is likely linked with the reduction of ovarian hormone production^15^. In agreement, ovariectomy, i.e., the complete removal of the source of ovarian hormones, has a similar defeminisation effect on female growth as T-treatment in both geckos^7,15^ and iguanas^5,19^. In contrast to the previous studies, Testosterone females of *C. calyptratus* attained similar body sizes as Control females which were much smaller than all male treatment groups, even though their ontogenetic allometry in casque size departed from Control females towards Control males (Fig. 3C). Small size of Testosterone females might be due to the persistent effect of ovarian hormones on female growth. All Control and Testosterone females laid similarly one to five clutches of unfertilized eggs, indicating that the reproductive function of ovaries was not completely compromised by high levels of androgens in Testosterone females (Table 1, Supplementary Fig. 2).

Surprisingly, according to static allometry, relative head shape reflecting relative casque size, was comparable among treatment groups at the time when the growth of all experimental animals considerably decelerated (Fig. 2, Supplementary Fig. 1). The logical interpretation of the static allometry pattern is that all groups follow the same, steep allometry in casque size and that the relative size of the casque is just a consequence of a shift along the common allometric line (see e.g., analogous situations in sexual dimorphism in shape in other lizards and human beings^44,45^). Nevertheless, the ontogenetic allometries revealed that a shift along common allometric line is incorrect for the chameleon casque. Control males and females differ significantly in casque growth during the ontogeny with females having less steep ontogenetic allometry. This observation further demonstrates that static and ontogenetic allometries can be largely different^46^ and that conclusions based just on static allometries might be misleading. As male castration has no effect on casque growth when compared to Control males (Fig. 3B), the steeper ontogenetic allometry in casque growth in Testosterone females in comparison to Control females might imply a negative effect of ovarian hormones on casque growth. Veiled chameleons possess an XX/XY sex-determining system^47^ but their sex chromosomes are only poorly differentiated^48^. Genes linked to sex chromosomes could be responsible for the ontogeny of sexual dimorphism in the chameleons; however, the departure from female-typical casque growth in Testosterone females suggests that sexual differences in casque size are not completely linked to sex chromosomes, but that their development is at least partially influenced by hormones.

In conclusion, our experimental study on veiled chameleons, *C. calyptratus*, demonstrates that male-specific growth and casque size is unlikely mediated by male gonadal androgens. These observations are consistent with the hypothesis that male growth is more a neutral, set stage and females depart from this “neutral” trajectory in absolute body growth as well as in relative growth of casque size under the influence of ovarian hormones. The ontogenetic allometries also showed that following ontogeny is crucial for understanding the development of sexual dimorphism in body size and its proximate mechanism and that interpretations based solely on static allometries can be inaccurate.

## Supplementary information

Supplementary information.

Supplementary information 2.

Supplementary information 3.

## Data Availability

The entire dataset used in our analyses can be found in the electronic supplementary material (Figs. S1, S2; Table S1).

## References

[1] Kratochvíl L, Frynta D (2002). Body size, male combat and the evolution of sexual dimorphism in eublepharid geckos (Squamata: Eublepharidae). Biol. J. Linn. Soc. Lond..

[2] Cox RM, John-Alder HB (2005). Testosterone has opposite effects on male growth in lizards (*Sceloporus* spp.) with opposite patterns of sexual size dimorphism. J. Exp. Biol..

[3] Cox RM, Stenquist DS, Calsbeek R (2009). Testosterone, growth and the evolution of sexual size dimorphism. J. Evol. Biol..

[4] Starostová Z, Kubička L, Kratochvíl L (2010). Macroevolutionary pattern of sexual size dimorphism in geckos corresponds to intraspecific temperature-induced variation. J. Evol. Biol..

[5] Cox RM, Calsbeek R (2010). Severe costs of reproduction persist in *Anolis* lizards despite the evolution of a single-egg clutch. Evolution.

[6] Bonnet X (2011). Which proximate factor determines sexual size dimorphism in tiger snakes?. Biol. J. Linn. Soc..

[7] Kubička L, Schořálková T, Červenk AJ, Kratochvíl L (2017). Ovarian control of growth and sexual size dimorphism in a male-larger gecko. J. Exp. Biol..

[8] Adkins-Regan, E. *Hormones and Animal Social Behavior*. (Princeton University Press 2005).

[9] Golinski A, Kubička L, John-Alder H, Kratochvíl L (2014). Elevated testosterone is required for male copulatory behavior and aggression in Madagascar ground gecko (*Paroedura picta*). Gen. Comp. Endocrinol..

[10] Rhen T, Ross J, Crews D (1999). Effects of testosterone on sexual behavior and morphology in adult female leopard geckos, *Eublepharis macularius*. Horm. Behav..

[11] Cox, R. M., Skelly, S. L., Leo, A. & John-Alder, H. B. Testosterone regulates sexually dimorphic coloration in the eastern fence lizard, *Sceloporus undulatus*. *Copeia* 597–608 (2005).

[12] Schořálková T, Kratochvíl L, Kubička L (2018). Female sexual attractiveness and sex recognition in leopard gecko: Males are indiscriminate courters. Horm. Behav..

[13] Schořálková T, Kratochvíl L, Kubička L (2017). Temporal organization: A novel mechanism of hormonal control of male-typical sexual behavior in vertebrates. Physiol. Behav..

[14] Schořálková T, Kratochvíl L, Kubička L (2018). To fight or mate? Hormonal control of sex recognition, male sexual behavior and aggression in the gecko lizard. Horm. Behav..

[15] Starostová Z, Kubička L, Golinski A, Kratochvíl L (2013). Neither male gonadal androgens nor female reproductive costs drive development of sexual size dimorphism in lizards. J. Exp. Biol..

[16] Kubička L, Starostová Z, Kratochvíl L (2015). Endogenous control of sexual size dimorphism: Gonadal androgens have neither direct nor indirect effect on male growth in a Madagascar ground gecko (*Paroedura picta*). Gen. Comp. Endocrinol..

[17] Kubička L, Golinski A, John-Alder H, Kratochvíl L (2013). Ontogeny of pronounced female-biased sexual size dimorphism in the Malaysian cat gecko (*Aeluroscalabotes felinus*: Squamata: Eublepharidae): A test of the role of testosterone in growth regulation. Gen. Comp. Endocrinol..

[18] Pollock NB, Feigin S, Drazenovic M, John-Alder HB (2017). Sex hormones and the development of sexual size dimorphism: 5α-dihydrotestosterone inhibits growth in a female-larger lizard (*Sceloporus undulatus*). J. Exp. Biol..

[19] Cox CL, Hanninen AF, Reedy AM, Cox RM (2015). Female anoles retain responsiveness to testosterone despite the evolution of androgen-mediated sexual dimorphism. Funct. Ecol..

[20] Darwin, C. R. The descent of man and selection in relation to sex. (John Murray 1871).

[21] Measey GJ, Hopkins K, Tolley KA (2009). Morphology, ornaments and performance in two chameleon ecomorphs: is the casque bigger than the bite?. Zool..

[22] Rieppel O (1981). The skull and jaw adductor musculature in chameleons. Rev. Suisse Zool..

[23] Ligon JD, Thornhill R, Zuk M, Johnson K (1990). Male-male competition, ornamentation and the role of testosterone in sexual selection in red jungle fowl. Anim. Behav..

[24] Setchell JM, Jean Wickings E (2005). Dominance, status signals and coloration in male mandrills (*Mandrillus sphinx*). Ethol..

[25] Schmidt, W. *Chamaeleo calyptratus, the Yemen Chameleon*. (Natur und Tier – Verlag 2001).

[26] Andrews RM, Donoghue S (2004). Effects of temperature and moisture on embryonic diapause of the veiled chameleon (*Chamaeleo calyptratus*). J. Exp. Zool. Part. A.

[27] Andrews RM (2005). Incubation temperature and sex ratio of the veiled chameleon (*Chamaeleo calyptratus*). J. Herpetol..

[28] Krysko K, Enge K, King F (2004). The veiled chameleon *Chamaeleo calyptratus*: A new exotic lizard species in Florida. Fla. Scientist.

[29] Kummrow MS, Gilman C, Mackie P, Smith DA, Mastromonaco GF (2010). Noninvasive analysis of fecal reproductive hormone metabolites in female veiled chameleons (*Chamaeleo calyptratus*) by enzyme immunoassay. Zoo. Biol..

[30] Diaz RE (2015). The veiled chameleon (*Chamaeleo calyptratus* Duméril and Duméril 1851): A model for studying reptile body plan development and evolution. Cold Spring Harb. Protoc..

[31] Pinto, B. J. *et al*. The transcriptome of the veiled chameleon (*Chamaeleo calyptratus*): A resource for studying the evolution and development of vertebrates. *Dev Dyn*. **248**, 702–708 (2019). 10.1002/dvdy.20 30839129

[32] Andrews RM (2008). Effects of incubation temperature on growth and performance of the veiled chameleon (*Chamaeleo calyptratus*). J. Exp. Zool. Part. A.

[33] Sosvorova L, Vitku J, Chlupacova T, Mohapl M, Hampl R (2015). Determination of seven selected neuro- and immunomodulatory steroids in human cerebrospinal fluid and plasma using LC-MS/MS. Steroids.

[34] Sathyapalan T (2017). Androstenedione and testosterone levels correlate with *in vitro* fertilization rates in insulin-resistant women. BMJ Open. Diabetes Res. Care.

[35] Hampl R, Putz Z, Stárka L (1990). Radioimmunologic determination of dihydrotestosterone and its value in laboratory diagnosis (In Czech). Biochem. Clin. Bohemoslov..

[36] St. Clair RC (1998). Patterns of growth and sexual size dimorphism in two species of box turtles with environmental sex determination. Oecologia.

[37] Kubička L, Kratochvíl L (2009). First grow, then breed and finally get fat: hierarchical allocation to life-history traits in a lizard with invariant clutch size. Funct. Ecol..

[38] Burnham KP, Anderson DR, Huyvaert KP (2011). AIC model selection and multimodel inference in behavioral ecology: some background, observations, and comparisons. Behav. Ecol. Sociobiol..

[39] Symonds MRE, Moussalli A (2010). A brief guide to model selection, multimodel inference and model averaging in behavioural ecology using Akaike’s information criterion. Behav. Ecol. Sociobiol..

[40] Bates D, Mächler M, Bolker B, Walker S (2015). Fitting linear mixed-effects models using lme4. J. Stat. Softw..

[41] R Core Team. R: A language and environment for statistical computing. R Foundation for Statistical Computing, Vienna, Austria, http://www.R-project.org/ (2013).

[42] Norris, D. O. & Carr, J. A. *Vertebrate endocrinology, 5 edition*. (Academic Press 2013).

[43] Zhou R, Bird IM, Dumesic DA, Abbott DH (2005). Adrenal hyperandrogenism is induced by fetal androgen excess in a rhesus monkey model of polycystic ovary syndrome. J. Clin. Endocrinol. Metab..

[44] Massetti F, Gomes V, Perera A, Rato C, Kaliontzopoulou A (2017). Morphological and functional implications of sexual size dimorphism in the Moorish gecko, *Tarentola mauritanica*. Biol. J. Linn. Soc..

[45] Kratochvíl L, Flegr J (2009). Differences in the 2nd to 4th digit length ratio in humans reflect shifts along the common allometric line. Biol. Lett..

[46] Pélabon C (2013). On the relationship between ontogenetic and static allometry. Am. Nat..

[47] Nielsen SV, Banks JL, Diaz RE, Trainor PA, Gamble T (2018). Dynamic sex chromosomes in Old World chameleons (Squamata: Chamaeleonidae). J. Evol. Biol..

[48] Rovatsos M (2017). Evolution of karyotypes in chameleons. Genes..

