# Supplementary material for: Little if any role of male gonadal androgens in ontogeny of sexual dimorphism in body size and cranial casque in chameleons
Source: Sci Rep. 2020 Feb 14;10:2673. doi: 10.1038/s41598-020-59501-6 (PMC7021717; doi:10.1038/s41598-020-59501-6)
Supplement: Supplementary file 1 — Supplementary information. [file 41598_2020_59501_MOESM1_ESM.pdf]

A

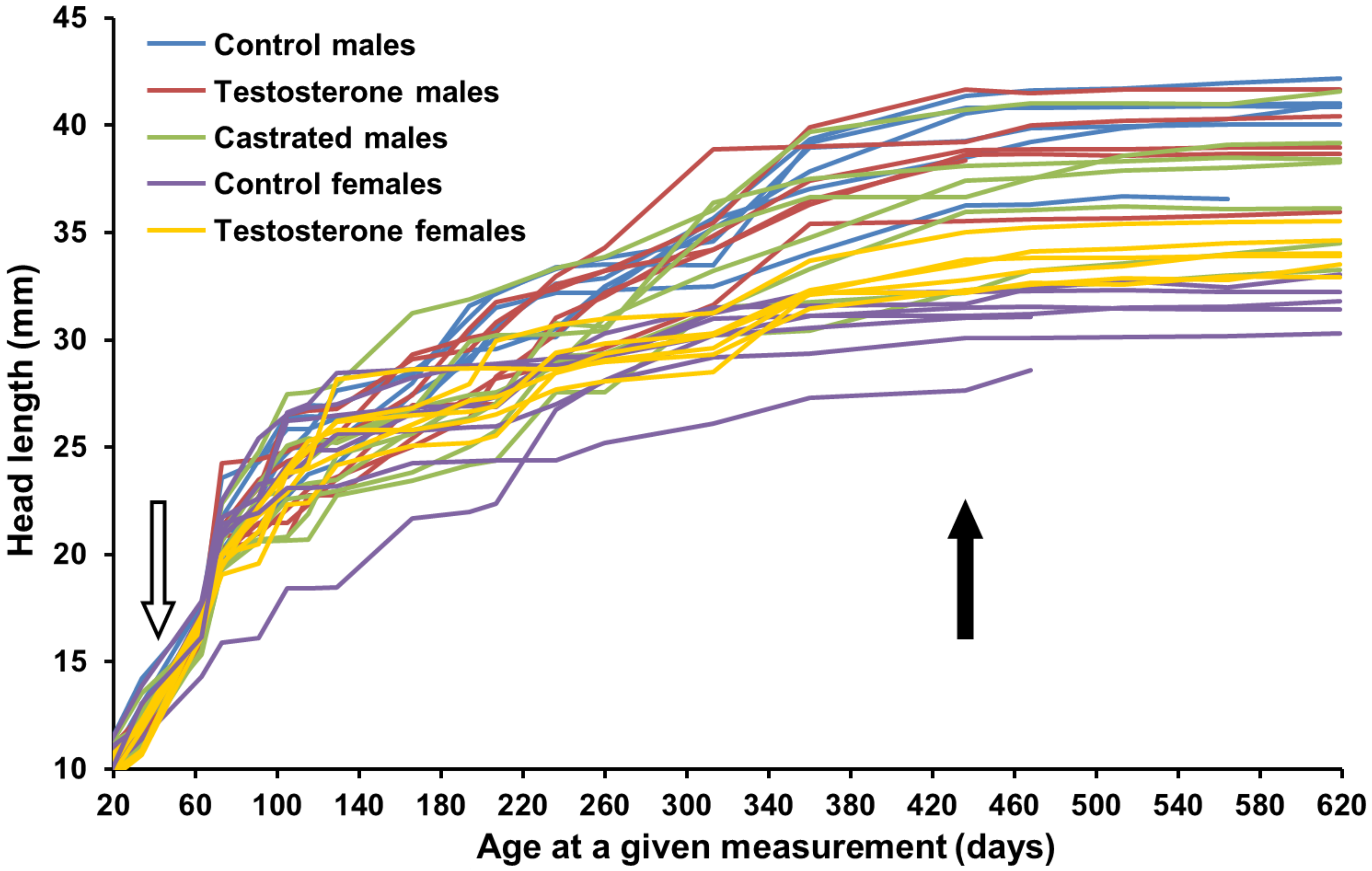

B

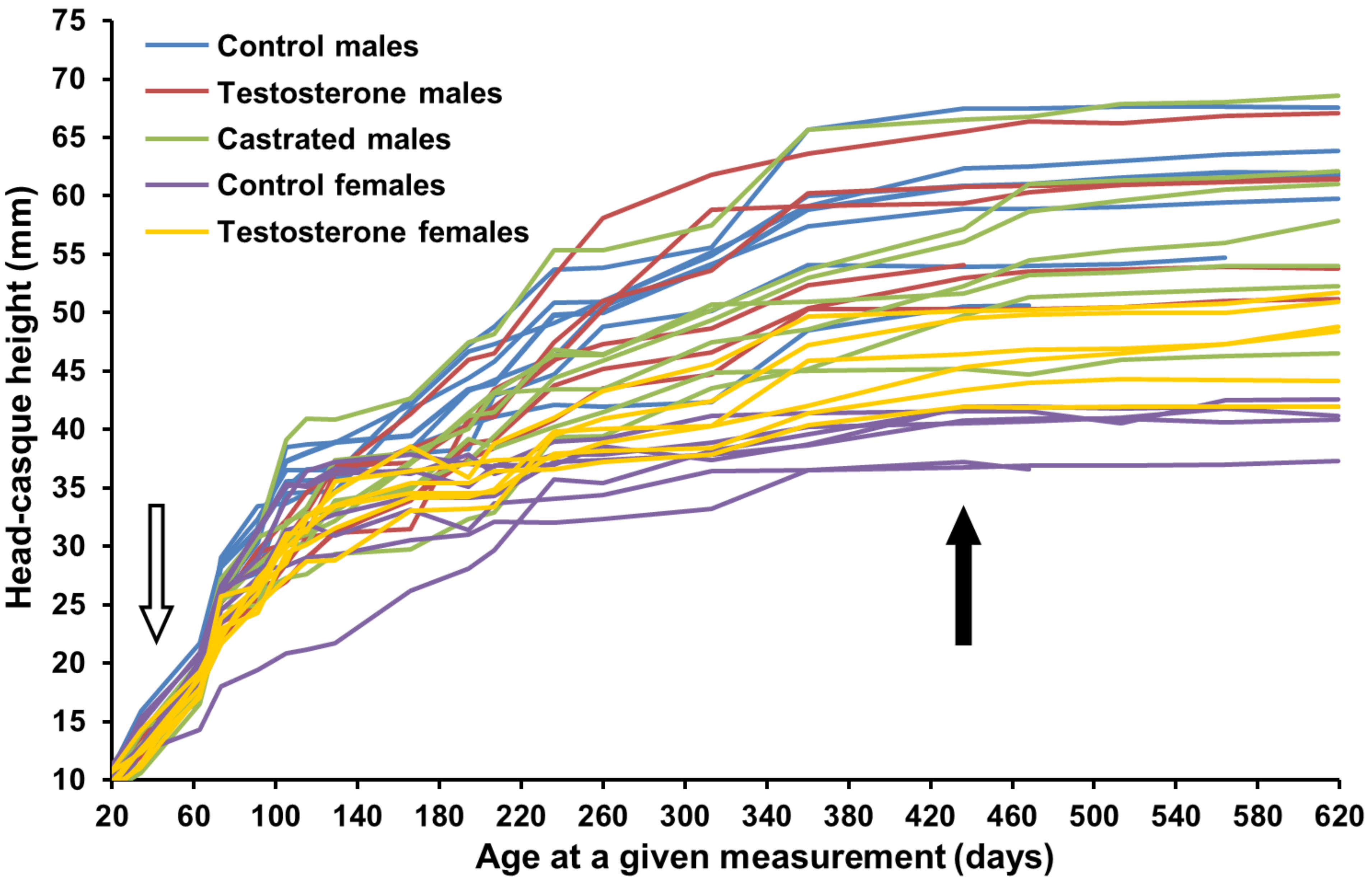

Supplementary figure 1. Growth trajectory of (A) head length and (B) head-casque height of each experimental veiled chameleon (*Chamaeleo calyptratus*, Chamaeleonidae, Iguania) during the whole experiment. Blue colour represent Control males, red colour Testosterone males (testosterone-treated castrated males), green colour Castrated males, lilac colour Control females and yellow colour Testosterone females (testosterone-treated females). Open arrows point to the beginning of manipulations, solid arrows point to the time when static allometry was examined.
