# Supplementary material for: Little if any role of male gonadal androgens in ontogeny of sexual dimorphism in body size and cranial casque in chameleons
Source: Sci Rep. 2020 Feb 14;10:2673. doi: 10.1038/s41598-020-59501-6 (PMC7021717; doi:10.1038/s41598-020-59501-6)
Supplement: Supplementary file 2 — Supplementary information 2. [file 41598_2020_59501_MOESM2_ESM.pdf]

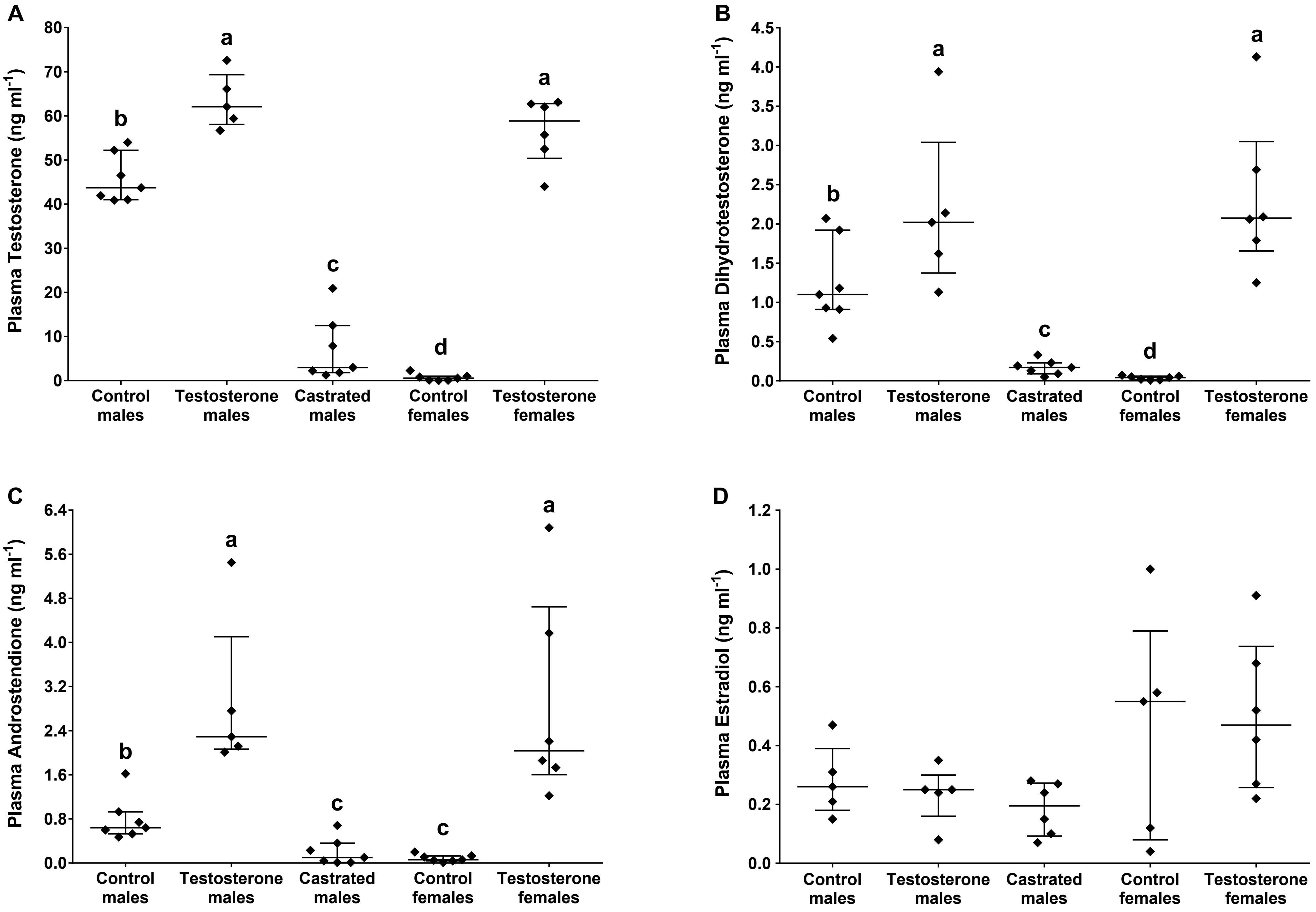

Supplementary Figure 2. Plasma concentration of testosterone (A), dihydrotestosterone (B), androstenedione (C) and estradiol (D) in experimental veiled chameleons (*Chamaeleo calyptratus*, Chamaeleonidae, Iguania). Testosterone males denote testosterone-treated castrated males, Testosterone females denote testosterone-treated females. For the comparisons of hormonal levels among treatment groups, the value of the hormone-specific detection limit was assigned to animals with levels below the limit of detection. Median and inner quartiles are shown. Letters denote statistically homogenous groups.
